# Vitamin D, exercise, and immune health in athletes: A narrative review

**DOI:** 10.3389/fimmu.2022.954994

**Published:** 2022-09-23

**Authors:** Clara Crescioli

**Affiliations:** Department of Movement, Human and Health Sciences, University of Rome “Foro Italico”, Rome, Italy

**Keywords:** Vitamin D, immune system, skeletal muscle, exercise, myokines, health, athletes

## Abstract

Vitamin D exerts important extra-skeletal effects, exhibiting an exquisite immune regulatory ability, affecting both innate and adaptive immune responses through the modulation of immunocyte function and signaling. Remarkably, the immune function of working skeletal muscle, which is fully recognized to behave as a secretory organ with immune capacity, is under the tight control of vitamin D as well. Vitamin D status, meaning hormone sufficiency or insufficiency, can push toward strengthening/stabilization or decline of immune surveillance, with important consequences for health. This aspect is particularly relevant when considering the athletic population: while exercising is, nowadays, the recommended approach to maintain health and counteract inflammatory processes, “too much” exercise, often experienced by athletes, can increase inflammation, decrease immune surveillance, and expose them to a higher risk of diseases. When overexercise intersects with hypovitaminosis D, the overall effects on the immune system might converge into immune depression and higher vulnerability to diseases. This paper aims to provide an overview of how vitamin D shapes human immune responses, acting on the immune system and skeletal muscle cells; some aspects of exercise-related immune modifications are addressed, focusing on athletes. The crossroad where vitamin D and exercise meet can profile whole-body immune response and health.

## Graphical Abstract

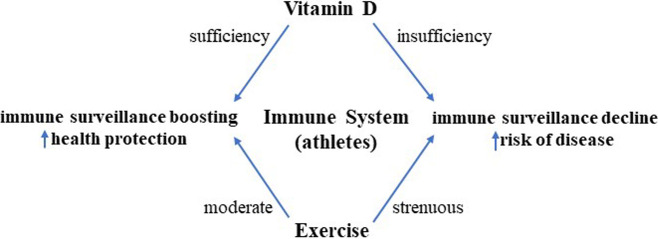

## Introduction

There is robust evidence of causative links between exercise, improved immunity, and disease prevention. Indeed, an optimal functioning immune system plays a central role in health maintenance, promoting a well-balanced defense against microorganisms or aberrant cells. In this light, exercise training is recommended as a multifaceted intervention for health (1, 2). Nevertheless, intense and prolonged exercise bouts seem to produce a temporary immunodepression, associated with a decreased host protection and, in turn, an increased risk of diseases, particularly infections, as documented by studies on athletes (3, 4). The human immune system is intensely shaped by exercise and by a variety of stimuli, such as stress, lack of sleep, general health status, environmental extremes (altitude), competition, and nutrients. Among others, vitamin D is a well-known regulator of the immune response, acting on several immune cell types, including macrophages, antigen-presenting cells (APC), dendritic cells (DCs), T cells, and B cells, which express vitamin D receptor (VDR), either constitutively or upon activation (5). Adequate levels of vitamin D are recommended to maintain immunity and prevent illness. Currently, overwhelming evidence suggests that D hypovitaminosis is similarly widespread in the general population and in athletes (6–9). In fact, albeit athletes are generally healthy subjects, many of them are vitamin D deficient, likely as a consequence of combined factors like poor/inadequate diet and sun underexposure (10, 11). A vitamin D-deficient athlete may be at an increased risk of potential problems like stress, fractures, respiratory infections, muscle injuries, and immune system depression. Remarkably, vitamin D remodels and strengthens immunity, not only acting directly on immune cells but also modulating the so-called immune ability of nonproperly working immune tissues, such as the skeletal muscle (12–14). Indeed, besides resident immune compartments, which exert inflammatory, protective, and reparative functions, the skeletal muscle can behave as a proper immune secretory organ, functioning as a checkpoint within a complex integrated network of immune-endocrine signals, malleable by exercise and vitamin D. Thus, the interplay between exercise and vitamin D status seems to play a pivotal role in immune health homeostasis.

This review aims to provide an overview of how vitamin D shapes human immunity, acting on both the immune system and skeletal muscle, and how it interplays with exercise to profile whole-body immune response, focusing on athletes. In the first part of the paper, some aspects of exercise-related modifications in the immune system are summarized with pros and cons.

## Exercise and the immune system: Is there a limit separating health and disease?

The importance of exercise on human health has been clarified since 400 BCE by Hippocrates, who stated, “…if there is any deficiency in food or exercise the body will fall sick.” It is recognized that the immune system is intensely modified by physical activity and exercise (15, 16).

A sedentary lifestyle is associated with an increased risk of comorbidities, including cardiovascular and metabolic diseases, cancer, neurodegeneration, and depression. These clustering diseases, reported as “diseasome of physical inactivity” (17), are essentially ascribable to immunity polarization toward T helper (Th)1/Th17-dominance and chronic inflammation, mediated by a plethora of immune/inflammatory active biomolecules, arising from immunocytes and adipocytes in consequence of inactivity-derived visceral fat accumulation (often accompanied by muscle mass decline) (18–20). The expanded adipose tissue, along with infiltrated resident macrophages, is recognized to be the main source of prototypic inflammatory Th1 cytokines, such as tumor necrosis factor (TNF)-α (21). Exercise-induced anti-inflammatory effects keep under control immune/inflammatory signaling with acknowledged benefits for health maintenance (22, 23). However, “too much” exercise, as much as experienced by athletes, likely does not support immunity, as addressed hereafter.

### Pros: An example from the elderly

In addition to restoring an optimal muscle/fat ratio, exercising is currently recognized to significantly decrease inflammation, protect against several immune/inflammatory diseases (24, 25), and the reduce morbidity or mortality rate in adulthood and older age by counteracting frailty and cognitive decline (26–29). Those positive effects are promoted by improvements in immune function and opposition to immune senescence, a biological age-related decline of immune surveillance, leading to higher susceptibility to infections, lower efficacy of vaccination, and higher risk of cancer (30–32). In the elderly, physical activity is associated with better immune response and better protection of the influenza vaccine (33, 34). In addition to the benefits of some age-related alterations, excellently summarized in a recent review (35), studies on young subjects document that exercise boosts the immune system by acting on circulating inflammatory cytokines and decreasing the secretion of several inflammatory cytokines, including TNF-α, interferon (IFN)-γ, interleukin (IL)-1β, IL-2, IL-6, and IL-8 (36, 37).

In early investigations, the anti-inflammatory effect was associated with an increased risk of infections due to exercise-induced immunodepression, as addressed hereafter.

Nowadays, regularly exercising is a well-recognized adjuvant of immune surveillance by balancing the Th1/Th2 ratio and opposing the interplay between inflammatory and oxidant processes, recently referred to as “oxinflammation” (38).

The decrease of reactive oxygen and nitrogen species (ROS and RNS, respectively) and the simultaneous increase of antioxidant defense—by potentiating enzymatic activity of catalase, superoxide dismutase, glutathione peroxidase—are examples of the multiple mechanisms involved in exercise-induced support to the immune system (38, 39). A recent meta-analysis on oxidative stress parameters concludes that training improves health-related outcomes, reducing the pro-oxidants/antioxidants ratio, regardless of the studied population, and independently of intensity/volume/type of exercise (40). Exercise significantly supports immune response by promoting immune cell recirculation from lymphoid tissues and their interchange with blood: intensity-dependent leukocytosis is followed by an increase in the number and redistribution of effector cells to peripheral tissues. Leukocyte recirculation following exercise likely depends on cell mobilization and demargination of previously circulating cells, driven by surface modifications of adhesion molecules, rather than de novo bone marrow release (41).

Even a single bout of exercise can promote the redistribution of natural killer (NK) and viral-specific T cells—thus limiting latent viral reactivation and reducing the antigenic load on T cells—and can prevent exhausted/senescent T-cell accumulation *via* apoptosis (35).

It has been suggested that the modifications to natural killer (NK) and T-cell trafficking promoted by exercise might potentially have important implications for health, i.e., by isolating mobilized lymphocytes for immune cell therapeutics (35, 41).

Acute exercising for less than 1 h transiently promotes the recirculation of B cells, NK cells, and CD8+ T cytotoxic lymphocytes exhibiting effector-memory phenotype, highly active in immune host defense (16, 42–44). Furthermore, during moderate exercise (lasting less than 1 h), stress hormones do not reach the high concentration needed to act as a suppressor of immunocyte activity (45). This transient effect results in immune surveillance boosting.

Conversely, intense/prolonged exercise is known for quite a long to increase circulating stress hormones, such as cortisol or catecholamines, which alter leukocyte trafficking and redistribution; in particular, catecholamines exert a greater impact on NK than T or B cells, in keeping with the density gradient of cell β receptors (41, 46, 47). Stress hormone-induced modifications to cell number, surface molecule expression, and cell deformation, found in different cell subsets, are greater with prolonged intense exercise, as exhaustively reported elsewhere (41).

Exercise-induced positive regulation of the immune system involves several mechanisms, including the qualitative shift from a Th1 to a Th2 response, the enhancement of mitochondrial function in peripheral blood mononuclear cells, and the regulation of immunometabolism toward more oxidative phenotypes (48–54). Thus far, albeit research in exercise immunology is still emergent and gaps in the knowledge exist, the summation of the effects induced by each bout of moderate exercise repeated over time significantly strengthens immune surveillance against pathogens, inflammatory disorders, and cancer cells by several mechanisms, collectively supporting the therapeutic potential of exercising (55, 56), as summarized in **Table 1**.

**Table 1:** The effect of effort intensity on immunity.

Effort intensity	Immune-related modification	Summation of effects
Moderate-to-vigorous regular exercise (less than 1 ) Stress hormones do not reach the concentration to suppress immune activity	+ macrophage antipathogen activity + immunosurveillance against cancer cells + immunoglobulins + anti-inflammatory cytokines + neutrophils + NK cells + T cells (particularly cytotoxic CD4+ T cells)	Immune defense activity enhancement, systemic inflammation decrease, diminished risk of illness
Prolonged and intensive endurance exercise Stress hormones reach the concentration to suppress immune activity	− macrophage function (altered MHC-II) − immunosurveillance against pathogens and cancer cells − neutrophil function − NK cell activity − salivary IgA output	Prolonged immune system alteration, systemic inflammation increase, increased risk of illness

The effects induced by moderate/vigorous exercise and prolonged/intensive endurance exercise on different immune components and cell types are compared, and the summation of the effect on immunity is depicted. The signs “+” and “−” indicate up- and downregulation, respectively.

Nevertheless, it is undeniable that heavy exertion as practiced by athletes may be associated with increased inflammation, oxidative stress, and increased risk of illness.

### Cons: An example from the athletes

The attention on immune response in athletes is currently high since heavy training workloads might turn to an immune dysfunctional response and increased risk of illness.

This phenomenon gives rise to questions on a possible edge separating the immune-depressive from the immune-boosting effect of exercise, particularly in athletes (57, 58).

The pioneering studies on changes in basic immune cell counts and function evidenced profound perturbations of leukocyte subsets linked to endeavor-related stress (57, 59–62).

Indeed, after prolonged/intensive endurance exercise, critical alterations in immunity biomarkers—salivary immunoglobulin (Ig) A output (suppressed), the function of NK cells, neutrophils, T cells, and B cells (reduced), expression of major histocompatibility complex II (MHC-II) in macrophages (downregulated), just to mention some—persist for hours to days, expose the athletes to illness higher risk (16, 63–68), in primis to an increased risk of upper respiratory tract infections (URTI) (61, 62, 69–76). **Table 1** summarizes the effects induced by prolonged/strenuous exercise. Indeed, overexercise suppresses MHC-II expression and negatively impacts macrophages’ ability to present the antigen to T lymphocytes, further impairing immune surveillance (77).

The relationship between the risk of URTI and exercise intensity in humans mostly emerged from self-reported sickness logs and was substantially confirmed in animals, albeit mechanistic experimental studies are often not immediately translated to humans, considering the difficult comparison across species due to the high variability of exercise protocols or adaptation (78–80).

The drastic reduction in lymphocyte number and function is observed within 1-2 h after exercise, a timeframe known as an “open window” similar to a break in immune surveillance as represented by a J-curve model (81). To date, this hypothesis has been argued and it is still under debate (68).

Over time, exercise immunology has received growing attention, and investigators have clarified that exercise-dependent immunity modulation specifically mirrors intensity, duration, and type of the effort, with different responses to acute/chronic, and moderate/vigorous regimens (differentiation criteria: 60% intensity threshold of oxygen and heart rate reserve, 60 min duration threshold).

In fact, exercise in a moderate regimen on a regular basis can decrease illness incidence by dampening inflammation and infections, as previously addressed (57, 82). Accordingly, consistent results from several randomized clinical trials show exercise-reduced URTI incidence and duration: summarizing, at least 5 days/week of aerobic exercise (from 20 min) can decrease by 43% the number of days with URTI vs. sedentary habits (exercising less than 1 day/week), as recently reviewed in an exhaustive paper on this topic (56, 83, 84). This result persists after adjustment of confounders, such as age, gender, education level, marital status, and mental stress (56, 57). The comparison between heavy and moderate exertion, such as marathon races and 30/45 min walking, respectively, supports the hypothesis that the perturbation of immune function specifically reflects the extent of stress experienced by the exerciser (56).

Athletes undergoing repeated heavy exertion cycles, i.e., in proximity to competitions, often experience concomitant stressors such as traveling, nutrition changes, sleep deprivation, and mental stress, all together merging in reduced immune surveillance, which, in turn, associated with the higher illness of respiratory tract, skin, digestive and genitourinary tract (58, 85). Albeit pure cause–effect relationships between heavy exertion and risk of diseases (either infective or not) have not yet been clarified, some chief organizations, such as the International Olympic Committee and the International Association of Athletic Federation, have introduced surveillance programs to prevent and manage this important problem (58, 86–88).

Thus, consensus statements with the ultimate goals of achieving performance and maintaining athlete’s health provided some key guidelines (4, 58, 89).

Remarkably, exercise stress represents such a challenge for the immune system, requiring biosynthetic and oxygen bioavailability to promptly reprogram and support effector cell metabolism and production of specific mediators, like cytokines, involved in the inflammatory response.

Indeed, intensely trained athletes show important alterations in the bioactive lipidome and proteome, like metabolites from lipid super pathways (oxylipins) or immune-related proteins, largely involved in immune cell chemotaxis and migration, mediating organ cross-talk during inflammatory responses (90–92).

With the development of high-resolution omics technologies, the recent hypothesis is that the transient immune dysfunction in the “open window” is due to a significant decrease in cell metabolic capacity during recovery immediately after intense exercise bouts, rather than being a general immune depressing response (93–95).

In this scenario, the multiomics approach highlights the importance of nutritional interaction on immune modifications in response to exercise. Of several factors, vitamin D status is highly critical, considering that this molecule can control whole-body immunity, affecting the immune system and the immune activity of skeletal muscle.

## Vitamin D and athletes: Dialoguing with the immune system

The diet of athletes should provide sufficient nutrients and micronutrients—proteins, carbohydrates, minerals, and vitamins—to meet their energy needs and maintain at best their immune health (4).

Even short-term deficiencies from dietary restrictions, often aimed to rapidly reduce athlete’s weight while continuing hard training, immediately turn into impaired immune surveillance (96–99). Furthermore, larger increases in circulating stress hormones and greater immune perturbation have been reported in athletes exercising in a carbohydrate-depleted state (96).

Vitamin D is a well-recognized upregulator of immunity, and matters arise from the observation that D insufficiency/deficiency is a common feature in athletes from different sports disciplines, including dancing, taekwondo, running, jockeying, and weightlifting (100–106).

The explanation for this widespread D inadequacy is likely due to different factors. First, ultraviolet (UV)B sunrays insufficient exposure, which is the main source of vitamin D, in addition to the diet (few foods naturally contain it) or vitamin D-fortified foods, as previously reported (107). The effectiveness of vitamin D endogenous synthesis seems to be affected by several factors, including latitude, season, atmospheric pollution, type of sport, indoor/outdoor training, lifestyle, sunscreen use, skin pigmentation (dark-skinned people need about 10-time longer sun exposure due to melanin concentration), albeit contradictory data are reported on this topic (108). Regardless of the cause, vitamin D hypovitaminosis is acknowledged in the global athletic population and attracts growing attention.

Studies on vitamin D inadequacy among athletes often are focused only on performance, as this molecule seems to act as a “performance enhancer, although conclusive data on this topic are still missing (109).

Instead, concerns should be addressed about general health rather than limited to performance, considering the tight control exerted by vitamin D on some important functions, broadly affecting health in all individuals, including athletes.

Vitamin D, behaving as a typical steroid hormone or as a micronutrient with rapid mechanisms, exerts pleiotropic effects *via* interaction with vitamin D receptor (VDR), virtually expressed by every human tissue (110, 111).

In addition to homeostasis regulation in bone, which is the classical tissue target of this molecule, it is well recognized that vitamin D significantly impacts the inflammatory status, which, in turn, is acknowledged as the common link in several noxious conditions, including infections, joint degenerative diseases, and disturbance of metabolism, to mention some (112–115).

Unfortunately, the ability of vitamin D to modulate the immune response can be listed among the main mechanisms underlying its anti-inflammatory effects.

Albeit immunocytes are considered nonclassical target cells of vitamin D, almost all types of immune cells, including CD4+ and CD8+ T cells, B cells, neutrophils, APCs, like macrophages, and dendritic cells (DCs), express vitamin VDR, which upon ligand binding modulates cell number and function (116). The multifaceted effects on the different immune cell types are extensively reported in the literature; essentially, they converge in promoting a shift from the Th1/Th17 inflammatory subset to protolerogenic dominance, in association with enhancement of T regulatory (Treg) cells and impairment of APC. Vitamin D signaling, indeed, ensures the suppression of proinflammatory status, downregulating T cells and cytokines like IL-2, IL-6, IL-8, IL-12, tumor growth factor (TGF)-β, IFN-γ, IL-17, and IL-21, simultaneously enabling Treg subset expansion with increased production of protolerogenic mediators, such as IL-4, IL-5, IL-13, IL-10, and CCL2 (117–121).

Vitamin D-dependent inhibition of DC differentiation from monocytes, antigen processing, and antigen presentation decrease—due to the downregulation of costimulatory molecules/major histocompatibility complex (MHC)-II-complexed antigen—and IL-10 upregulation, further supporting protolerogenic signals (122).

In particular, vitamin D can modify DC morphology to a more adherent spindle shape, and surface markers drive the cells to a less mature/more tolerogenic phenotype, in association with a decrease in cluster of differentiation (CD) 80, CD86 (costimulatory molecules), and CD54 (adhesion molecule), in addition to MHC-II downregulation, whereas the expression of CCR5 (chemokine receptor), DEC205 (antigen-uptake receptor), F4/80 (macrophage marker), and CD40 increases, resulting in a general downregulation of antigen presentation function (123, 124).

The overall effect likely acts as a “balance” of the inflammatory response evoked by long/high-intensity exercise (above 80% VO2max, 120 min) characterized by proinflammatory cytokine rise, i.e., IL-6, IL-1, IL-8, and TNF-α, as reported, whereas it seems to merge with the effect of short-term/moderate exercise (50%–75% VO2max, 45–60 min), associated with an expansion in T-cell-derived Th2 mediators, as IL-4 and IL-10.

Interestingly, IL-10 promotes long-lasting antigen-specific T-cell anergy and plays a driving role for type 1 T regulatory (Tr1) cells, the cell subset known to be critical for maintaining tolerance to self and nonself antigens in humans and animals, in the presence of APC, as emerged from *in vitro* experiments (125–128).

In line with previous *in vitro* investigations reporting IL-10 increase and IFN-γ reduction in peripheral blood mononuclear cells after vitamin D, recent *in vivo* experimental studies on atheroprotection document that vitamin D added to dexamethasone significantly promotes IL-10 by DC as well as other APC, thus establishing IL-10 network of lymphoid and myeloid immune cells, and simultaneously reduces Th1 response by inhibiting IFNγ-producing CD4+ and CD8+ T cells (129).

Noticeably, vitamin D-induced mechanisms underlying the transition from proinflammatory IFN-γ+ Th1 cells to suppressive IL-10+ cells seem primed by wide-epigenetic T-cell remodeling, which promotes VDR expression and enzyme cytochrome P450 family 27 subfamily B member 1 (CYP27B1) activation by autocrine/paracrine mode, leading to Th1/Th17 program repression (*via* STAT3, c-JUN, and BACH2) and IL-10 enhancement (*via* IL-6–STAT3 signaling), as recently shown by elegant research in coronavirus disease 2019 (COVID-19) patients (130).

Subsequent studies are encouraged to verify whether similar/different vitamin D-dependent mechanisms occur in immune adaptation to exercise.

**Figure 1:**
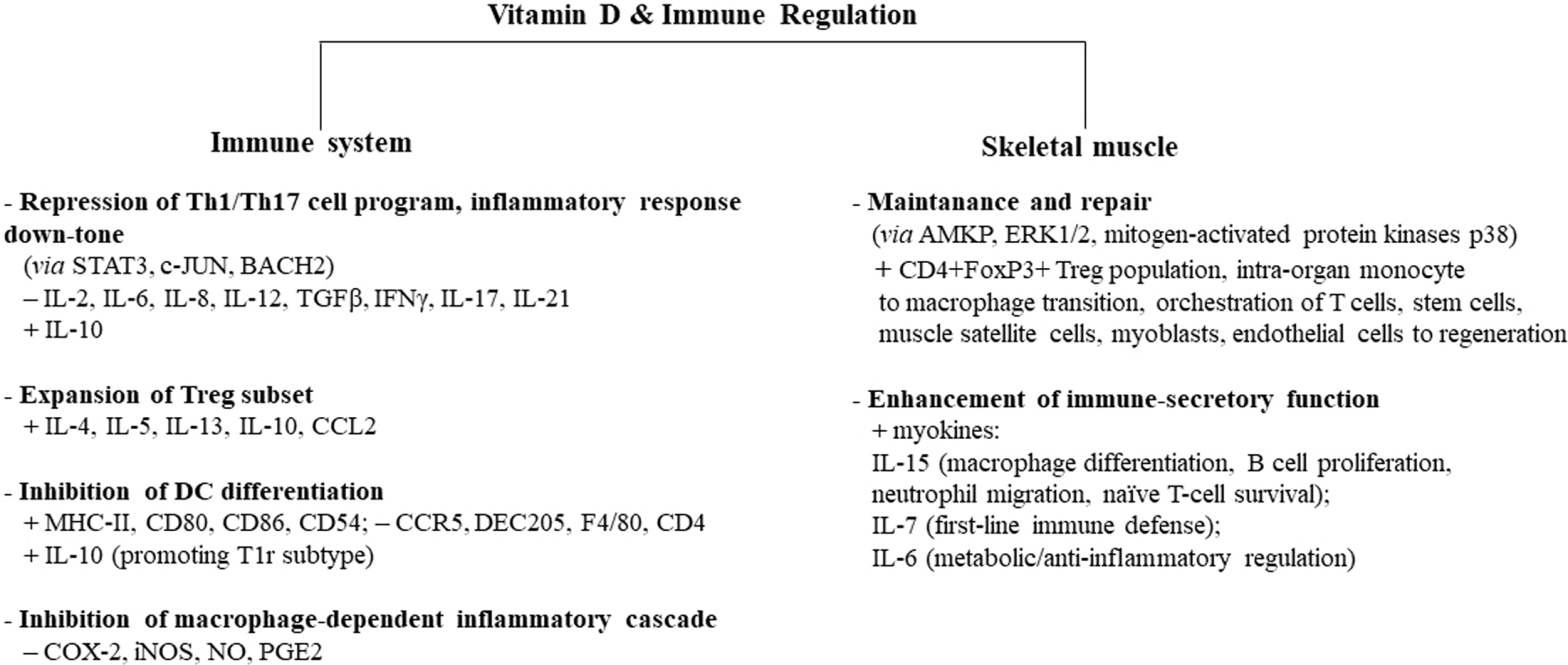
Vitamin D-induced immune regulation of the immune system and skeletal muscle. Vitamin D controls inflammation and promotes tolerogenic status, acting on several types of circulating immune cells, skeletal muscle cells, and intraorgan immune cells. Some cell types, biomediators, and signaling paths/molecules mainly involved in this process are indicated. Similar immune regulatory mechanisms are promoted by moderate exercise, while intense/prolonged exercise leads to a decline in immune surveillance, recalling the immunodepression allowed by sedentary status. Adequate vitamin D levels can downtone exercise-induced inflammatory-like response and converge to exercise-induced immunosurveillance boosting, with protective effects on whole-body health. Overtraining athletes in hypovitaminosis D can be at higher risk of infectious and noninfectious diseases. The signs “+” and “−” indicate up- and downregulation, respectively. AMPK, 5′ adenosine monophosphate-activated protein kinase; ERK1/2, extracellular signal-regulated kinase 1/2; Tr1, type 1 T regulatory cells; MHC-II, major histocompatibility complex II; STAT3, signal transducer and activator of transcription 3; c-JUN, transcription factor Jun; BACH2, transcription regulator protein broad complex-tramtrack-bric a brac and Cap'n'collar homology 2; COX-2, cyclooxygenase 2; iNOS, inducible nitric oxide synthase; NO, nitric oxide; PGE2, prostaglandin E2.

As already addressed, regular short-term/moderate-intensity exercise strengthens the immune system by increasing macrophage activity, which is further potentiated by the vitamin D effect on monocytes. Of note, vitamin D induces macrophage and epithelial cells to produce cathelicidin, a protein with marked antimicrobial activity, able to improve macrophage bacterial capacity involved in host-first-line defense (131–134). Furthermore, vitamin D impairs macrophage inflammatory cascade by targeting cyclooxygenase 2 (COX-2) and inducible nitric oxide synthase (iNOS) and, therefore, reducing nitric oxide (NO) and prostaglandin (PG)E2 (135). **Figure 1** summarizes the main effects induced by vitamin D in different types of immunocytes.

The higher production of cathelicidin and defensin (another host-defense peptide) induced by vitamin D along with its anti-inflammatory action would promptly reduce the cytokine storm during infection by COVID-19 (136). Indeed, recent investigations in COVID-19 patients document the usefulness of vitamin D administration due to the protective effects against mortality and intensive care unit admission (137, 138).

Adequate vitamin D status combined with the practice of exercise seems to promote positive outcomes in COVID-19, albeit the research on this specific topic is still in its infancy (139).

It has been reported that sex-dependent dimorphism in vitamin D metabolism likely explains the greater immune vulnerability to perinatal infections observed in male vs. female fetuses/neonates due to the testosterone-induced decrease of cathelicidin gene, through the inhibition of alpha hydroxylase CYP27B1, the enzyme necessary for D bioactive form (140). It would be interesting to extend this kind of investigation into adulthood to verify whether similar mechanism(s) may underlie some sex-dependent differences in response to infections (including COVID-19), which are also seen in athletes and are frequently mistreated (141, 142).

In B cells, vitamin D inhibits proliferation and immunoglobulin production similarly to heavy exertion, whereas repeated bouts of moderate-intensity exercise enhance B-cell proliferation.

Since B and T cells, macrophages, and DCs can self-synthesize vitamin D, a kind of “local” anti-inflammatory effect occurs within infiltrated target tissues (5, 143). Exercise upregulates VDR expression in T cells regardless of exercise-induced T-cell mobilization (144), therefore enhancing the anti-inflammatory loop. Thus far, vitamin D can impact both innate and adaptive immunity with a decisive anti-inflammatory profile. While this effect was initially simplistically considered immunosuppressive, the current concept focuses on the exquisite modulating role of vitamin D toward tolerogenic homeostasis (145).

Conversely, low vitamin D levels are associated with deficits in immune surveillance, including lower salivary IgA and increased risk of long-lasting respiratory infection, as observed in elite athletes (11). Indeed, hypovitaminosis D-shaped modifications in the immune system often converge on and amplify heavy exertion-induced effects.

Furthermore, vitamin D deficiency is described in the pathophysiology of Th2-driven allergic diseases such as asthma, in which the lower hormone levels are associated with IL-4, IL-5, IL-9, and IL-13 deregulation, increase in asthma markers (IgE and eosinophil), and more severe clinical disease manifestation, as reported (146–149).

To date, vitamin D deficit-dependent damage is not limited to the immune system but highly impacts the function of skeletal muscles; since this tissue exhibits important immunocompetent capacity, whole-body immune surveillance is further compromised.

## Vitamin D and the immunity of skeletal muscle

Skeletal muscle is a nontraditional target tissue of vitamin D and is finely regulated by this molecule at several levels. According to experimental and human studies, insufficient vitamin D levels and VDR deletion cause critical muscular dysfunctions (150–153).

In fact, skeletal muscle development, myocyte differentiation, muscular volume, tissue functional maintenance, and physical performance are processes tightly dependent on the intact vitamin D/VDR system, as confirmed by studies in humans with VDR mutations or in VDR knockout (VDRKO) mice (151–153).

Lower levels of vitamin D are associated with a significant reduction in muscle fiber size and atrophy (mainly of type II fiber), and overall, determine muscular defects in energy handling (as insulin resistance), plasticity, and contraction, in the general population and in athletes as well (154, 155).

Conversely, higher vitamin D levels are reported to be linked with lower injury rates and improved sports performance (156).

The beneficial effects of vitamin D on skeletal muscle function are related to the fine-tuned regulation exerted at the cell level through VDR interaction, albeit, in the past, the presence of this receptor in human muscle was questioned (157). To date, VDR is mainly detected in fast-twitch muscle fibers (committed to rapid actions) and expressed at different levels in human isolated cells, depending on the cell fusion stage (upregulation upon myotube formation) (150, 158–161).

Intramyonuclear VDR concentration is directly associated with vitamin D serum level, suggesting that the circulating vitamin D/muscular VDR system, plays a pivotal role in the integrity of skeletal muscle, rather than hormone deficiency alone (158). In line with this hypothesis, VDR/D deficit promotes a series of biomolecular alterations, including increased oxidative stress and decreased antioxidant activity, converging in muscle deterioration and ending in atrophy (107, 162).

Type II fiber atrophy significantly ameliorates with vitamin D, as documented in biopsies from vitamin D-deficient patients before and after the treatment with the hormone (163). Vitamin D helps faster recovery from muscle injury and inflammation after high-intensity exercise (164, 165), whereas vitamin D-deficient athletes show a delayed recovery. Upon VDR expression increase, the intracellular signaling cascade involved in repair processes—such as 5′ adenosine monophosphate-activated protein kinase (AMPK), extracellular signal-regulated kinase (ERK)1/2, mitogen-activated protein kinases p38—is activated and interferes with proinflammatory molecule genes (166). Generally, it can be stated that vitamin D affects almost all stages of the myogenic program toward regeneration, also acting on satellite cells. Due to myocyte’s ability to uptake vitamin D from the bloodstream, skeletal muscle tissue accumulates this hormone and acts as a functional reservoir, ready to release it upon blood-level decline. Interestingly, regularly exercising maintains and enhances this functional feature (167).

Exercise is a well-known strategy against muscle wasting and atrophy, not only because it counteracts mass loss but because it exquisitely regulates the mitochondrial function and the internal immune component, both critical for muscle integrity maintenance during stress, as shown by multiomics analysis in astronauts during spaceflights (168).

Of note, exercise- and vitamin D-induced signals converge in the dynamic remodeling of mitochondria, promoting correct genomic reprogramming and skeletal muscle cell remodeling (169).

Beyond those beneficial effects, it is mandatory to highlight the function of vitamin D in maintaining the immune-secretory function of skeletal muscle, which is closely in line with the topic of this review.

Nowadays, the renewed and proven concept is that skeletal muscle is a proper secreting organ with immunoregulatory function. Indeed, upon contraction the muscle releases many trophic/immunoreactive small peptides, the myokines, which can control the function of nearby or distant organs, acting in an autocrine/paracrine/endocrine fashion, as recently reviewed in an exhaustive paper (170). Those factors, before their full identification, were referred to as the “work factors” or “exercise factors”, to clearly state that their release occurs exclusively upon muscular contraction and work (170). Currently, more than 650 myokines are identified by the proteomic analysis of the muscular secretory profile, which is constantly updated (171). Among this plethora of biomolecules, some myokines drew attention due to their ability to modulate the immune response, introducing a novel view of immunity-muscle crosstalk, which was previously considered to be a unidirectional route, with muscle being under immune system control (and not vice versa).

Indeed, like other tissues, skeletal muscle has its resident immune cell population to warrant the regenerative potential and tissue homeostasis. The CD4+FoxP3+ Treg population is the main subset infiltrating damaged muscle upon to micro- or macroinjuries, as well as during exercise, and drives muscle regeneration and satellite niche fate; intraorgan monocyte to macrophage conversion plays a pivotal role in orchestrating T cells, mesenchymal stem cells, muscle satellite cells, myoblasts, and endothelial cells towards muscle regeneration or pathogen clearance (94, 162, 172).

Fiber damage due to different injuries, including contusions, strains, hyperextensions, avulsions, or ruptures, promptly activates neutrophils resident in skeletal muscle to release within the microenvironment high concentrations of inflammatory factors necessary for repair (173, 174).

Albeit several types of leukocytes, such as mast cells, neutrophils, eosinophils, and lymphocytes, participate in the repair/regeneration, the monocyte/macrophage population controls all stages of this process (175). Indeed, after neutrophils, macrophages represent the second subpopulation reaching the injured areas (peak at 3 to 6 days and persisting 2 weeks after extensive damage), gradually shifting from a phagocytic to pro-myogenic phenotype, from M1 to M2 macrophages, respectively (172). The shift in macrophage phenotype orchestrates the time of myogenic sequence, supporting first cell proliferation and migration, while delaying differentiation, and then facilitating alignment and fusion (176, 177). During regenerative processes, soluble molecules as growth factors, cytokines, and prostaglandins regulate immune and muscle cell communications, but interestingly, close cell-to-cell contacts between myogenic cells and macrophages occur *via* adhesion molecules, macrophage pseudopodial extensions, and myogenic cell cytoplasmic protrusions (174, 178, 179). T cells show a delayed response, roughly 4 days after the initial damage (172, 180).

Adequate vitamin D levels support the function of the immune intraorgan component: its role, generally considered pro-tolerogenic, is, indeed, to dampen the damaging effects of cell stress and immune response during excessive or chronic reactions, and, in this view, this molecule is defined “pro-survival” (181). Furthermore, exercise-induced production of some myokines, in particular, IL-6, IL-7, and IL-15, by skeletal muscle cells, gives the muscle an “immune-like feature” and the capacity to impact leukocyte subset trafficking, immune cell function, and inflammation (35, 182).

Interleukin-6 is the prototypic myokine, the first one and most extensively studied. Differently from systemic proinflammatory “bad IL-6”, deriving from immune cells and adipocytes, muscular “good IL-6” is transiently released in the blood during exercise (up to a 100-fold increase, depending on intensity) and exhibits an unquestionable anti-inflammatory and metabolic profile (183).

Exercise-related pulsatile release of IL-6 promotes the anti-inflammatory macrophage subset (M2-like), involving suppressor of cytokine signaling 3 (SOCS3) ablation, and IL-1 receptor antagonist (IL-1ra) and IL-10, resulting in overall downregulation of inflammatory responses (52, 184). Interleukin-6 likely plays a central role in exercise-induced leukocytosis and late lymphopenia mediated by cortisol, as shown by IL-6 infusion in athletes (52).

In humans, IL-6 is known to counteract TNFα production and signaling from monocytes (185, 186).

Furthermore, IL-6 behaves as an energy biosensor in conditions of energy shortage/demand, such as during physical exercise, enhancing hepatic glucose production, and promoting fat oxidation (187).

Thus far, the myokine IL-6 likely characterizes exercise adaptation, as it is involved in long-term beneficial effects, related to an exercise-training reduction in abdominal fat and anti-inflammatory actions (188). Vitamin D can enhance the biological effects of IL-6, as shown by the improved metabolic function observed in vitamin D-deficient trained men after a single intramuscular injection of vitamin D, which was associated with a significant rise of IL-6 1 h after resistance exercise (189). The lack of modification in inflammatory parameters is likely due to the short duration of the treatment and the use of a single dose.

Interleukin-6 output from human skeletal muscle cells maintained in nutrient restriction, to mimic energy-demanding conditions such as postexercise, was significantly increased after the treatment with a VDR agonist (162). Conversely, the addition of a VDR agonist to human muscle cells challenged by a strong proinflammatory environment significantly counteracted inflammation-induced intracellular cascade underlying Th1-type chemokine release (190). Thus far, vitamin D modulation seems to be beneficial with prometabolic or anti-inflammatory effects, depending on the microenvironmental needs of skeletal myocytes.

Muscle-derived IL-15 regulates macrophage differentiation, B-cell proliferation, neutrophil migration, and naïve T-cell survival (191). This myokine tightly cooperates with vitamin D, promoting the conversion into the active hormone, the upregulation of VDR, and the induction of cathelicidin (192, 193).

IL-7 also plays a pivotal role in first-line immune defense; the age-dependent decline of this myokine can be counteracted by exercise and by vitamin D, which can help to restore aberrant IL-7-dependent signal, i.e., occurring in immunosenescence or autoimmune processes (194–196).

Thus far, vitamin D is a good enhancer of some exercise-induced “immune” adaptations of the skeletal muscle. The main immune regulatory effects of vitamin D on intraorgan immunocytes and myocytes are depicted in **Figure 1**.

## Conclusions

Vitamin D insufficiency/deficiency is so extensive across the world to ideally meet the criteria for the statement “pandemic”, and, in addition to the general population, it seems to affect the athletic population as well.

Athletes are thought to be in good health almost by definition, considering that many human diseases are tightly related to sedentary behavior and inflammation; importantly, the latter is a well-recognized bridge linking different (and clustering) illnesses.

Nevertheless, the condition of overexercising, too often experienced in several sports disciplines, exposes athletes to a higher risk of inflammation and, consequently, a higher risk of diseases.

This phenomenon is essentially related to the modulation of the immune system by exercise, which can enhance or decrease human immune surveillance, essentially depending on the athlete’s experienced effort. In this scenario, vitamin D status plays a critical role in immune health, as possible exercise-induced detrimental effects might merge with the poor immune health status determined by hypovitaminosis D. Conversely, vitamin D adequacy counteracts inflammation, enhancing the immune defense and shaping the immune response of skeletal muscle, which is recognized to be a proper secreting organ with immune-like features.

Thus far, screening for vitamin D status would be mandatory in the athletic population as well. This topic still represents a hot topic in literature, as important issues regarding vitamin D determination and supplementation, representing a possible strategy to limit the “pandemic” hypovitaminosis, are still far from being translated into practice, as thoroughly reported by the Consensus statement from the 2nd International Conference on Controversies in Vitamin D (197). The lack of discussion on these aspects is among the limits of this review, which does not include sex-dependent differences in immune response or in vitamin D levels, or cardiovascular features. Nevertheless, recalling the attention to the crossroad where exercise and vitamin D are likely to meet to shape immune health hopefully will help to bring further attention to an issue that is highly significant for athletes and the general population’s wellbeing.

## Author contributions

The author confirms being the sole contributor of this work and has approved it for publication.

## Conflict of interest

The author declares that the research was conducted in the absence of any commercial or financial relationships that could be construed as a potential conflict of interest.

## Publisher’s note

All claims expressed in this article are solely those of the authors and do not necessarily represent those of their affiliated organizations, or those of the publisher, the editors and the reviewers. Any product that may be evaluated in this article, or claim that may be made by its manufacturer, is not guaranteed or endorsed by the publisher.
